# Development and validation of a new standardised data collection tool to aid in the diagnosis of canine skin allergies

**DOI:** 10.1038/s41598-019-39630-3

**Published:** 2019-02-28

**Authors:** N. D. Harvey, S. C. Shaw, S. C. Blott, J. A. Vàzquez-Diosdado, G. C. W. England

**Affiliations:** 1 School of Veterinary Medicine and Science, The University of Nottingham, Leicestershire, LE12 5RD United Kingdom; 2 UK Vet Derm Ltd, 16 Talbot Street, Whitwick, Leicestershire LE67 5AW United Kingdom

## Abstract

Canine atopic dermatitis (cAD) is a common hereditary clinical syndrome in domestic dogs with no definitive diagnostic tests, which causes marked morbidity and has a high economic impact internationally. We created a novel questionnaire for Labrador (LR) and Golden retriever (GR) owners to evaluate canine skin health with respect to clinical signs of cAD. 4,111 dogs had fully completed questionnaires (2,803 LR; 1,308 GR). ‘Cases’ (793) had a reported veterinary diagnosis of cAD, and ‘controls’ (1652) had no current or past clinical signs of cAD and were aged >3 years. Remaining dogs (1666) were initially categorised as ‘Other’. Simulated annealing was used comparing ‘Cases’ and ‘Others’ to select a novel set of features able to classify a known case. Two feature sets are proposed, one for use on first evaluation and one for dogs with a history of skin problems. A sum for each list when applied to the whole population (including controls) was able to classify ‘Cases’ with a sensitivity of 89% to 94% and specificity of 71% to 69%, respectively, and identify potentially undiagnosed cases. Our findings demonstrate for the first time that owner questionnaire data can be reliably used to aid in the diagnostic process of cAD.

## Introduction

The most common cause of pruritic (itchy) allergic skin disease in dogs is canine atopic dermatitis (cAD). Approximately 10% of all dogs are affected in the developed world and it is becoming increasingly recognised globally^1^. cAD has a strong genetic predisposition and causes recurrent to continuous episodes of scratching, rubbing, chewing and licking resulting in inflamed and damaged skin, often complicated by bacterial and yeast secondary infections resulting in potentially severe skin lesions^1,2^. cAD has detrimental effects on the dog’s quality of life, through pain and discomfort, interruption of normal behaviour such as playing and sleeping and can be an expensive and distressing condition for the owner to manage^3^. Due to its international prevalence and impact there has been a push towards development of advanced therapeutics, many of which are high cost, to try and better manage the condition and reduce pruritus. This has led to the creation of the first biologic therapy approved by the European Union for use in veterinary medicine^4^.

The causes of cAD are complex, thought to arise from interactions between the dog’s genetics, epigenetics, immune response and allergen exposure in the dog’s environment^5,6^. The most important allergens in cAD are thought to be house dust mites^7^, storage mites, pollen and mould spores. Food allergies causing cutaneous signs (food-associated atopic dermatitis) may be a separate or co-existing condition^8^. Studies of heritability of cAD suggest that roughly half of dogs born to atopic parents will develop cAD themselves^7^. Several different breeds have been shown to be more likely to develop cAD, including Labrador and Golden retrievers, English Springer spaniels, Hungarian Vizlas, Basset hounds, Rhodesian Ridgebacks, Boxers, Chinese Shar Pei, West Highland white terriers, Bull terriers, French Bulldogs, Bichon Frisé and Tibetan terriers^8,9^. Breeds are known to have breed-related phenotypic variations in atopy suggesting the influence of breed-specific factors, although it cannot be assumed that these are genetic factors as they could be related to different ways in which different breeds are housed or managed^10^. However, there are certain key features of the disease that are common across all breeds such as affected paws, ventral abdomen, ears and axillae (armpits)^10,11^.

cAD is diagnosed by exclusion of other causes, with no definitive laboratory or clinical test available. This process is time-consuming and complicated as dogs may present with a variety of clinical signs (i.e. different areas of the body may be affected and varying severity), many of which may also be caused by other skin conditions. Once a diagnosis has been reached, treatment options need to be tailored to the individual depending on clinical signs, and these can change over time, requiring regular re-evaluation^12^.

Several authors have attempted to define diagnostic criteria for canine atopic dermatitis. Willemse^13^ used a minor and major criteria approach similar to that used in human medicine, providing a solid base for the recognition of cAD, which provided the case definition for many later clinical and pharmaceutical developments. These founding criteria were later altered as it was recognised that the inclusion of positive allergy tests as positive criteria for cAD was inappropriate as many unaffected animals show positive test results (Prélaud *et al*., 1998, described in^14^). More recently, using a large group of atopic dogs recognised by board-certified dermatologists, simplified and statistically validated criteria were developed by Favrot and colleagues^15^. Comparing these three diagnostic criteria, the original criteria of Willemse were highly specific (80.2%), but of poor sensitivity (49.3%), Prélaud improved the sensitivity (74.3%) with a specificity of 68.4% and Favrot further improved sensitivity (85%) and specificity (79%). This reveals that, at this time, the existing diagnostic criteria are unable to replace clinical evaluation, with many allergic dogs being undiagnosed by these methods. Despite some differences, most of the criteria share commonalities by requiring for example: age of onset of clinical signs to be below 3 years; affected front paws; affected ears (but not margins); cortico-steroid responsiveness and chronic/recurrent dermatitis or yeast infections.

Clinical examination scales, such as the CADESI-4, have been developed for scoring the severity of cAD associated lesions for use in clinical trials^16^, however these tools are not suitable for widespread epidemiological use, due to the need to be administered by a veterinary professional in the clinical setting and do not evaluate pruritus directly. The Edinburgh Pruritus Scale, a validated scoring system for pruritus severity^17^, suggests that carefully designed questionnaire style methods could be employed to gather information about a dog’s skin health direct from the owners prior to, or *in lieu* of clinical evaluation. Certain factors involved in the risk of developing cAD are still unclear, for example whether there is a sex predisposition and in what direction^18^, or an accurate prevalence of cAD in different breeds^15^. The development of a data-gathering tool that could be used on a large scale would aid the evaluation of factors such as these on cAD risk and severity. It is also likely that estimates of cAD prevalence based upon dogs receiving a veterinary diagnoses are underestimates, as not only could some dogs with more minor signs not be taken to the vets for diagnosis, but some dogs may be treated for individual flare ups and have them recorded as such in their medical history without it being recognised as atopy^1^. A tool that can be used to collect clinical data of relevance to atopic dermatitis direct from owners could help to improve prevalence estimates.

The aim of this study was to develop a questionnaire for completion by dog owners that could be used to identify dogs with diagnoses of cAD and other skin conditions, as well as suitable controls, including those who may have undiagnosed cAD. Such a questionnaire would enable large-scale epidemiological evaluation of the skin health of populations of dogs, without the need for clinical evaluations. Further, if such a tool were shown to be valid, it could be used in a veterinary context as a standardised method of data collection in the evaluation, and re-evaluation of cAD, to aid diagnoses of newly presenting cases, and to assist in identifying or adjusting treatment regimens.

Novel selection criteria were identified through simulated annealing of the questionnaire responses to identify diagnosed-cases with maximum sensitivity, exclude controls and those with non-cAD conditions. Following this, to validate the developed tool, we describe the performance of the classification and characterise the skin health and allergy signs present in each group of dogs identified by the new criteria.

## Results

In total, there were 4,479 responses to the carefully tailored owner-friendly questionnaire (see Supplementary Table S1 for all questions asked) in the 4 months during which it was active, of which 60 were automatically disqualified, 417 were labelled partial, and 4,002 were labelled as completed. Of the 417 labelled partial, 58 had not completed the registration page, so their responses were deleted, as they provided no consent to store their details. Ninety-four partials had registered their dog but had not answered any of the questions in the questionnaire, leaving 148 responses where the questionnaire was partially completed; with the remaining 114 having gone on to complete the questionnaire in a separate response. Of the 4,002 completed questionnaires, 42 dogs were in the dataset twice with their owner having completed the questionnaire on two occasions for reasons unknown; the first entry for each duplicate was removed, retaining only the second entry. The final dataset consisted of 4,111 responses, of which 3,963 (96%) were complete and 148 (4%) were partially completed.

### Subjects

Of the 4,111 useable responses 2,803 (68%) were Labrador retrievers (1432 M:1371 F) and 1,308 (32%) were Golden retrievers (668 M: 640 F). A total of 88% of the dogs were Kennel Club registered, 9% were not and 3% were not known. The majority of dogs (92%) were from the United Kingdom, 5% were from the United States, 1% from Canada, 1% from Ireland and less than 1% from other countries. The majority of responses were from people informed of the project by The Kennel Club (62%), with 15% through Facebook, 6% had simply said they were emailed about it and the remainders were via other sources or didn’t answer. The vast majority (84%) of dogs were described as pets, 8% were trained as gundogs, 3% were show dogs, 3% breeding dogs, and the remainder were ‘Other’ types of dog (2%) or working dogs (1%) (see Supplementary Table S2 for full number breakdowns).

The mean age of dogs in the sample was 6.1 years (SD ± 2.7), with 18 dogs that were less than 1 year of age and 11 dogs aged 17. With regard to neuter status, 66% (2732) of dogs in the sample were spayed or castrated, whilst 33% (1346) were intact, 1 was ‘Not Known’ and 32 were missing responses. The median age at neutering was 12 months.

### Diagnostic criteria

Using logic-based criteria (Supplementary Fig. S2, Supplementary Table S3) 40% of the sample was designated as a Control, 19% as a Case with a veterinary diagnosis of cAD, and 41% Other (Table 1).

**Table 1:** Sample breakdowns by breed and sex for dogs designated as Cases, Controls and Other.

Type	Labrador	Golden Retriever	Total
Case	575 (327 M: 248 F)	218 (115 M: 103 F)	793 (442 M: 351 F)
Control	1120 (513 M: 607 F)	532 (242 M: 290 F)	1652 (755 M: 897 F)
Other	1,108 (592 M: 561 F)	558 (311 M: 247 F)	1666(903 M: 763 F)

Simulated-annealing applied to the Case and Other data identified two new sets of potential diagnostic questions from the questionnaire that could classify the known cases (Table 2). The full list of 13 identified features contained questions that could only be answered for dogs with a past history of skin problems (full list), whilst retaining only those features that could be answered based purely upon presenting clinical signs left 8 questions (reduced list). As all of the features were binary in nature, a simple sum could be made and the appropriate cut-off for prediction maximising the sensitivity was selected.

**Table 2:** The full and reduced list of features selected for classification of known-cases of canine atopic dermatitis.

Full List	Reduced List
Responds to treatment that isn’t steroids	Current or past signs of abnormal itchiness
Has known food allergies	Current or past areas of abnormal skin
Has had yeast skin infections	Front paws itch
Dogs skin improves on exclusion diet	Collar region itches
Dog receives flea control	Armpit area itches
Current or past signs of abnormal itchiness**	Elbow skin appears abnormal
Current or past areas of abnormal skin*	Muzzle skin appears abnormal
Front paws itch	Skin on back paws appears abnormal
Collar region itches	
Armpit area itches	
Elbow skin appears abnormal	
Muzzle skin appears abnormal	
Skin on back paws appears abnormal	

^*^Abnormal skin: red, patchy, hairless, rough, swollen or discoloured. ^**^Abnormal itchiness: frequent and recurrent rubbing, licking, chewing or scratching of the same area. All features were binary in nature, with 1 indicating a positive answer.

**Figure 1:**
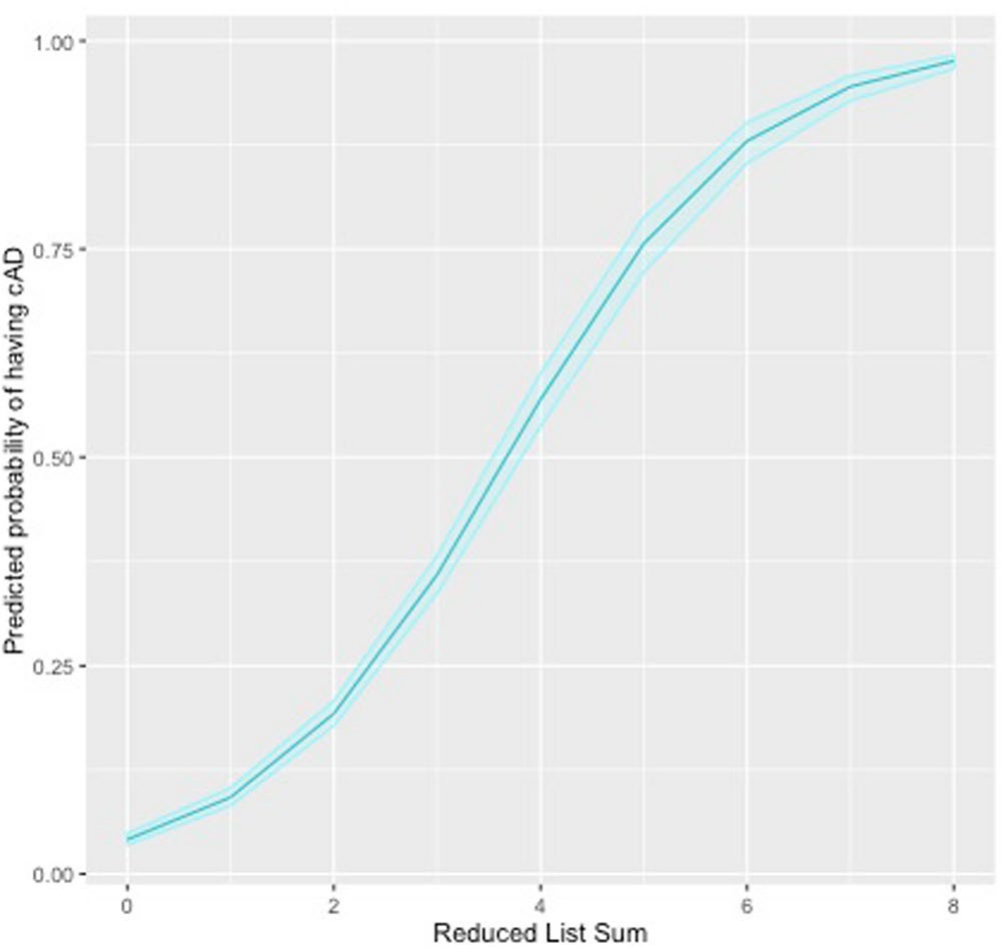
Predicted probability of being a Case having atopic dermatitis as a function of a dogs reduced list sum. The 95% confidence intervals are shown around the predicted probability, calculated using profiled log-likelihood.

The full list classified cases with a sensitivity of 94% and specificity of 69% (overall accuracy 74%) if 3/13 features were answered positively (PPV for a known case rate 42% and NPV 98%). The reduced list, with a cut off of 2/8 features being answered positively, was able to classify cases with a sensitivity of 89% and specificity of 71% (overall accuracy 74%, PPV for a known-case 42%, NPV 97%). The summed scores were linearly associated with a dog being a case or not, with dogs with higher summed scores having increased chances of being a case. For every additional feature answered positively in the full list, the dogs odds of being a case increased by 1.13 (GLM: OR 2.13, Z 29.19, p < 0.001) and for the reduced list by 1.35 (GLM: OR 2.35, Z 27.94, p < 0.001, Fig. 1). Both methods have a ‘false positive’ rate of just below 6/10, however it is worth noting that only 0.2% Controls were classified a Case by the reduced list, and 0.4% by the full list (out of 1,652). The ‘false positives’ are therefore not dogs with healthy skin, but dogs with skin problems that resemble cAD that could be thus far undiagnosed cases and would warrant further clinical evaluation.

In order to investigate how similar the non-cases classified as a ‘false positives’ are to the known cases on all facets of their skin health, the ‘false positives’ identified by the reduced list (the first that would be used when a dog presents with clinical signs) were then evaluated as a separate group to known cases, controls and dogs with ‘Other’ skin conditions. We refer to these as Potential Cases, as they had not received a diagnosis of cAD at the time of the questionnaire but have been predicted to be cases.

Comparison of the answers provided for Cases (with existing veterinary diagnosis), Controls, Potential Cases and Others allow us to establish the construct validity of the questionnaire for identifying dogs with and without the disease by ascertaining whether our findings met expectations based upon what is currently known about cAD. For example, Cases would be expected to score highest on the modified-Edinburgh pruritus scale, and Controls the lowest, whilst Potential Cases should score similarly to Cases if they are indeed dogs with undiagnosed cAD.

### Skin Health

#### Questions regarding dogs with cAD only

Considering just those dogs diagnosed with cAD (Cases), 41.5% had not tried an exclusion diet, but of those who had, 44.7% said it had seen their dog’s skin improve, and 55.3% said it had not helped. The majority (79.6%) of respondents did not know whether their dogs’ relatives had the same or similar skin conditions, with 10.6% saying No and 7.1% saying Yes. A total of 18.5% had not tried steroids, but of those who had, 89.5% said it had seen their dogs skin improve, and 10.5% said it had not helped. 25.3% of owners had not tried medication other than steroids. Of those who had, 89.7% said other medication had resulted in improvement, and 10.3% said it had not helped. In regard to seasonality, 50.4% of Cases had a recognisable seasonal pattern to their skin problems. Of these, the majority (63.2%) were worse in the summer, with 31.2% worse in the spring/autumn and only 5.6% worse in the winter. In terms of age of onset, 81% of Cases reportedly began showing signs when the dog was 3 or younger, and 19% when aged 4 or over. The most common period for cAD to begin was between 6m-3yrs (56% of dogs).

#### Skin conditions in controls, cases, potential cases and other

Four groups of dogs were compared on their skin health responses: known-cases; controls; potential cases (non-cases classified as cases using the feature lists) and ‘other’ (dogs with some form of skin condition classified as a non-case using the feature list).

With regards to co-morbidity with cAD, Cases had significantly higher proportion of other diagnosed skin problems than any other group (Tables 3 & 4). Just over half (51%) of Cases had also received a diagnosis of otitis externa (ear infections), compared to 5% of Controls, 42% of Others and 34% of Potential Cases. A quarter (25%) of dogs with cAD and 11% of Potential Cases also had diagnosed food allergies. Considering differences between Potential Cases and dogs with a diagnosis of cAD (Cases), the Potential Cases were significantly less likely than diagnosed Cases to have a diagnosis of food allergies, yeast skin infections, bacterial skin infections, flea allergic dermatitis and otitis externa but more likely to have a diagnosis of ‘wet eczema’ (acute moist dermatitis) (Table 3). There was no difference in occurrence of mange between any group. Considering Potential Cases and Others, there was no significant difference in disease occurrence for flea allergic dermatitis and ‘wet eczema’, but the Potential Cases were significantly more likely than the Others to be diagnosed with food allergies, yeast skin infections and bacterial skin infections, and significantly less likely to be diagnosed with otitis externa.

**Table 3:** Logistic regression results for each type of skin co-morbidity incidence showing the statistical significance, odds ratio and 95% confidence interval for Cases and Others as compared to Potential Cases.

	Case			Other		
	p	OR	95% CI	p	OR	95% CI
Food Allergies	**<0.001**	**2.86**	**2.20–3.70**	**0.021**	**0.66**	**0.47–0.94**
Mange	0.960	1.02	0.56–1.85	0.637	0.85	0.45–1.64
Yeast skin infection	**<0.001**	**3.27**	**2.37–4.50**	**<0.001**	**0.34**	**0.19–0.60**
Bacterial skin infection	**<0.001**	**2.15**	**1.65–2.80**	**0.001**	**0.53**	**0.37–0.76**
Flea allergic Dermatitis	**0.001**	**2.21**	**1.37–3.54**	0.174	0.63	0.32–1.23
Ear infections (otitis ext erna)	**<0.001**	**2.02**	**1.67–2.45**	**0.001**	**1.4**	**1.15–1.71**
Wet eczema (acute moist dermatitis)	**0.013**	**0.67**	**0.49–0.92**	0.096	0.77	0.56–1.05

**Table 4:** Cumulative incidence (also known as incidence proportions) figures for each skin disease diagnosis received for the dogs in a self-selected study sample (n = 4,111) as reported directly by their owners.

Skin disease	Total	Disease incidence by breed^a^		Proportion of disease by sex^b^		Disease incidence within each study group^c^				Incidence outside of cases^d^
		Labrador	Golden retriever	Male	Female	Case	Potential Cases	Other	Control	
Atopic dermatitis	793	575	218	442	351	—	—	—	—	
%	19%	21%	17%	56%	44%	—	—	—	—	—
Food allergies	371	268	103	217	154	200	102	51	18	46%
%	9%	10%	8%	58%	42%	25%	11%	7%	1%	
Mange (uncharacterised)	59	41	18	32	27	20	24	15	0	66%
%	1%	1%	1%	54%	46%	3%	2%	2%	0%	
Yeast skin infection	214	159	55	115	99	139	59	43	1	35%
%	5%	6%	4%	54%	46%	18%	6%	6%	0%	
Bacterial skin infection	317	183	134	193	124	166	106	43	2	48%
%	8%	7%	10%	61%	39%	21%	11%	6%	0%	
Flea allergic dermatitis	90	53	37	43	47	49	28	13	0	46%
%	2%	2%	3%	48%	52%	6%	3%	2%	0%	
Ear infections (otitis externa)	1107	786	321	603	504	404	328	295	80	64%
%	27%	28%	25%	54%	46%	51%	34%	42%	5%	
Wet eczema (acute moist dermatitis)	265	125	140	164	101	69	120	69	7	74%
%	6%	4%	11%	62%	38%	9%	12%	10%	0%	

^a^The proportion of dogs in each breed with the disease in question.

^b^The proportion of dogs with the disease in question that are male or female.

^c^The proportion of dogs in each group with the disease in question.

^d^The proportion of the population that do not have a diagnosed atopic dermatitis that reported having the associated condition.

In addition to the diseases described in Table 4, 19 golden retrievers were reported in the free text boxes to have ichthyosis, whilst a further 19 dogs were reported to have dandruff/flaky skin, and 2 further dogs as having their skin turn black.

#### Skin questions relevant to all groups

**Figure 2:**
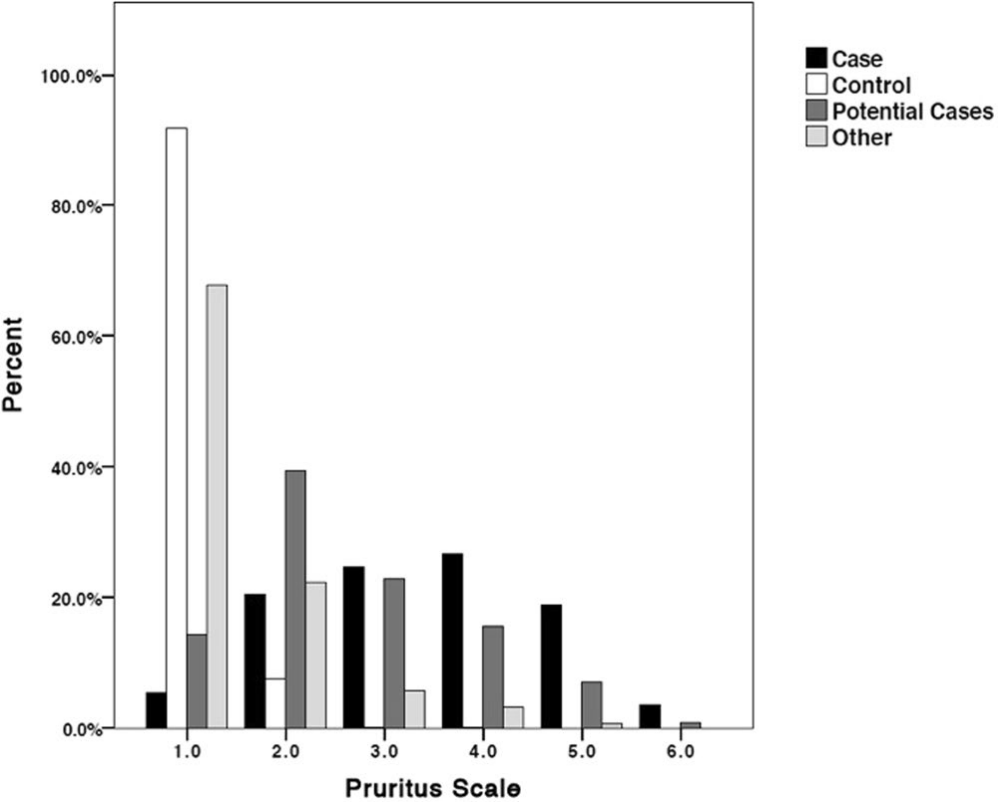
Edinburgh Pruritus Scale scores for each skin group. A score of 1 represents normal grooming behaviour and 6 represents almost continuous itching.

Exactly 50% (397) of Cases had been allergy tested, compared to 9% of Potential Cases, 3% of Other and 1% of Controls. Compared to Controls, Cases, Potential Cases and Other dogs were reported as having significantly more gastric issues (frequent loose stools) (GLM: p < 0.001, Wald = 44.59) and vomiting (GLM: p < 0.001, Wald = 23.02), but were not significantly different from each other for either variable. There was a significant difference in modified-Edinburgh Pruritus Scale scores for all skin groups (Fig. 2, ANOVA, df = 3, F = 1517.32, p < 0.001) [although it must be noted that Controls were classified as dogs whose owners answered ‘no’ to an earlier question indicating that they had had no “current or past signs of abnormal itchiness”]. Potential Cases scored similarly to Cases, but Cases had significantly higher mean pruritus scores (Tukey post-hoc mean difference 0.80, p < 0.001).

**Figure 3:**
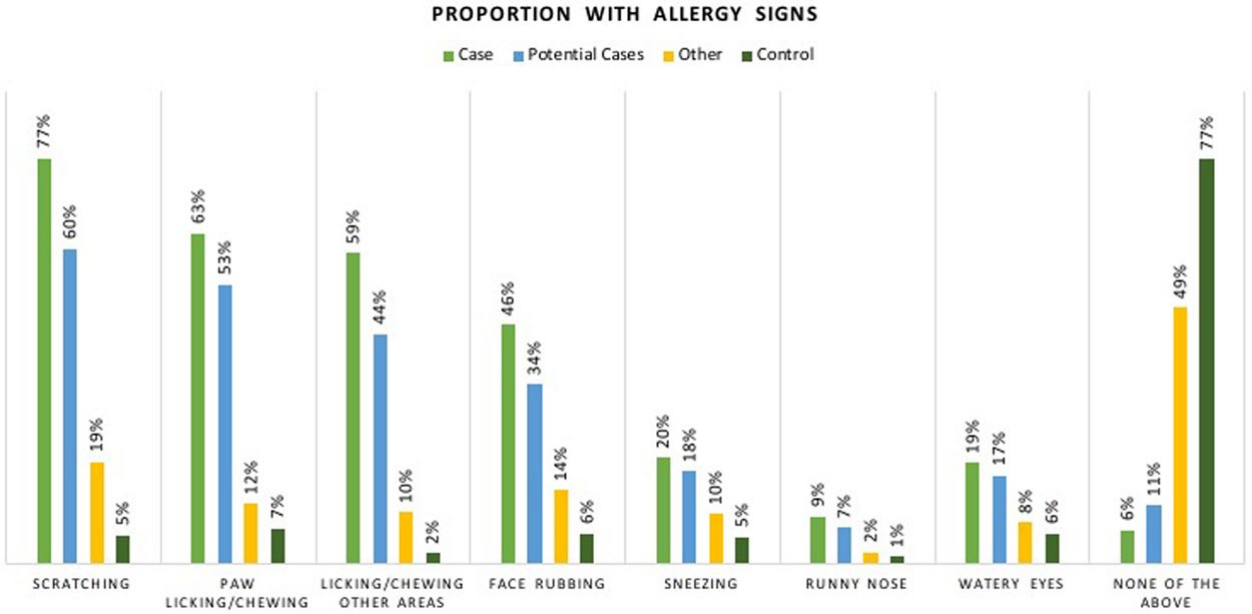
Percentage of dogs in each group that exhibit frequent and recurrent signs of allergies.

Amongst the Cases, 77% were reported as having had abnormal appearing skin now or in the past, compared to 69% Potential Cases and 20% of Others, whilst 88% of Cases and 86% of Potential Cases were reported as having had itchy skin now or in the past, compared to 22% of Others (no controls answered ‘yes’ here as answering ‘no’ to these were part of the selection criteria for a control). Cases and Potential Cases showed the highest proportion of allergy signs in terms of whether they could be seen to recurrently and frequently: scratch, paw lick/chew; lick/chew other areas; rub their face; sneeze; have a runny nose; or have watery eyes (Fig. 3). 60–70% of Potential Cases and Cases displayed abnormal levels of scratching, respectively, and paw licking/chewing was seen by 53–63% of these groups. 44–59% displayed abnormal levels of licking/chewing other body areas, and 46% of Cases and 34% Potential Cases repeatedly rubbed their faces. Comparatively, only 2–7% of Controls and 10–19% of Others were seen exhibiting these behaviours. It must be noted that these allergy signs were not used to classify the Potential Cases.

#### Questions relevant only to dogs with skin conditions

For dogs reported to have had a skin condition, 48% of Cases said their vet had suggested a diet trial, compared to 16% of Potential Cases, and 6% of Other.

Compared to dogs in the Other group, Cases and Potential Cases recurrently itched most on their fore paws (58% and 50% vs 5%), underbelly (51% and 35% vs 17%), hind paws (36% and 26% vs 5%) and armpits (axillae: 35% and 27% vs 2%) (Supplementary Fig. S3). Potential Cases only itched more than Cases in the collar region (22% vs 16% and 4% Others), whilst those in the Other group only itched with a high proportion (25%) on their ears (Cases 46%, Potential Cases 33%).

**Figure 4:**
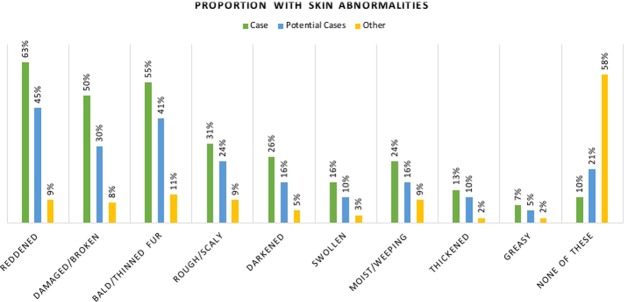
Proportion of dogs in each problem skin group that exhibited different types of skin abnormalities associated with atopic dermatitis.

With regards to abnormal and damaged skin, Cases and Potential Cases exhibited a much greater proportion of reddened, damaged, rough/scaly skin and bald/thinning fur than did dogs in the Other category (Fig. 4). Other dogs were least likely to exhibit every form of abnormal skin, and Potential Cases exhibited slightly to moderately fewer of all abnormalities than Cases with all differences statistically significant saving for ‘greasy’ skin (Supplementary Table S4).

**Figure 5:**
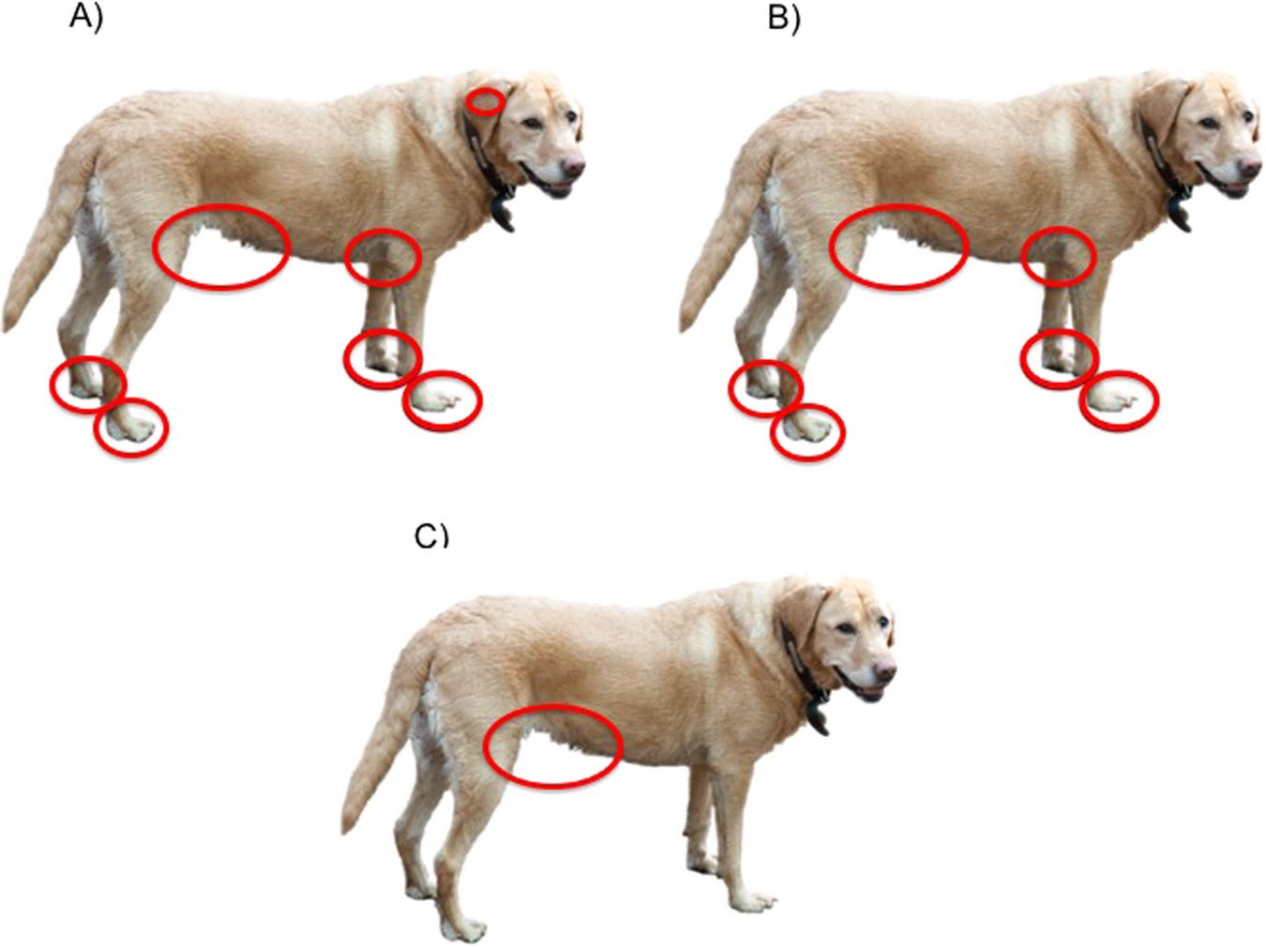
The most commonly affected areas of abnormal skin (where at least 20% of dogs were affected) for (**A**) dogs with a veterinary diagnosis of cAD, (**B**) dogs with no existing veterinary diagnosis of cAD but that may have cAD (Potential Cases) and (**C**) dogs with other skin conditions.

The main areas of the dogs’ bodies that were affected by the skin abnormalities shown in Fig. 5, for Cases and Potential Cases compared to Others were: fore paws (41% and 29% vs. 3%), hind paws (28% and 21% vs 1%), armpits (axillae: 36% and 23% vs 10%); underbelly (57% and 36% vs 27%) and elbows (14% and 18% vs 4%) (Supplementary Fig. S4 and Supplementary Table S5). Potential Cases had significantly fewer lesions than Cases in every region except for the elbows, collar region and back, but they had significantly more lesions than Others everywhere except for the inside of their ears, and their back (Supplementary Table S5). Although less affected, Potential Cases showed a similar pattern to Cases in the location of skin abnormalities by body area (Fig. 5).

Of the seven *a-priori* expectations that were tested in order to provide evidence for construct validity (whether a test measures what it reports to measure) of the questionnaire, all seven were met (Table 5).

**Table 5:** Expected associations between Cases, ‘false positives’, Controls and other groups if the Cases and ‘false positives’ were to reflect accepted clinical phenotypes of cAD.

Expectation	Based on	Met?
Cases should score highest on the modified-Edinburgh pruritus scale, whilst Potential Cases should score similarly to Cases.		Yes
Other skin conditions including ear infections, food allergies and yeast infections should have a higher disease incidence amongst Cases and Potential Cases than Controls and Others.		Yes
Acute moist dermatitis should have a higher disease incidence in Golden retrievers than Labradors	Wiles *et al*.^25^	Yes
Ear and yeast infections should have a higher disease incidence in Labradors than Golden retrievers	Layne & DeBoer^24^	Yes
Cases and Potential Cases should exhibit far greater recurrent signs of allergies than Controls and Others		Yes
Cases should have an itch and lesion distribution focused mainly on their fore and hind paws, axillae, inner ears, muzzle, eyes and ventral abdomen	Hensel *et al*.^11^	Yes (although eyes and muzzle were less affected)
Approximately 4/5 of Cases should have first developed clinical signs when under 3 years of age	Favrot *et al*.^19^	Yes

References are given where evidence for an expectation exists in the published literature. Construct validity for the questionnaire as identifying true cases of cAD is shown when these expectations are met.

## Discussion

The aim of this study was to develop a questionnaire for completion by dog owners with accompanying classification criteria that could identify dogs with diagnoses of cAD and other skin conditions, as well as suitable controls, and enable us to predict those dogs that may have thus-far undiagnosed cAD. During the 4-months that the survey was open we were able to recruit over four thousand dog owners, with 4,111 useable questionnaires. Epidemiological evaluation of the questionnaire answers revealed four groups of dogs: Cases (dogs with diagnosed cAD); Controls; ‘Potential Cases’ which may have undiagnosed cAD and Other (dogs with non-cAD skin conditions).

It must be acknowledged that there was no control over diagnosis in this study. The questionnaire, named the canine atopic dermatitis research and diagnosis questionnaire (cAD-RQ) relies upon owner report of veterinary diagnoses. As there is no standard diagnostic test for cAD, practitioners must rely upon presence and severity of a range of clinical signs and their own experience to make a diagnosis^7^. It is therefore possible that dogs may have been misdiagnosed due to similarities with other similar conditions. Therefore, the cAD-RQ is considered to evaluate cAD *sensu lato*; in the broad sense, due to the widest range of allergens. Control animals were over the age of 3 years and had exhibited no signs of skin disease.

To establish construct validity of the cAD-RQ as evaluating true cases of cAD, and identifying true potential cases, the results of this study were compared to what would be expected for dogs with clinically sound presentation. All of the findings presented here match well to what would be expected if our group of owner-reported Cases were true cases of cAD, providing support for the construct validity of the cAD-RQ as a tool for evaluating cAD via owner report.

The pattern of affected body areas for Cases and Potential Cases matched well to the pattern that would be expected for dogs with cAD^11^, with the most commonly affected areas being the front and back paws, axillae, inner ears and underbelly/groin. The muzzle and eye area would also be expected to be a commonly affected body region for cAD^11^, but here only Cases exhibited itching in the muzzle area for more than 20% of dogs. As with previous studies, the majority of Cases developed clinical signs when under 3 years of age; 81% of dogs here compared to 78% previously reported^19^, confirming that one fifth of Cases develop cAD when aged over 3. Further, Cases scored significantly higher on the modified-Edinburgh Pruritus Scale than any other group and had a significantly higher cumulative incidence than any other group for associated skin conditions including ear, yeast and bacterial infections and food allergies as would be expected^20^. Based upon these results there is good reason to believe that the dogs reported here as having cAD by their owners do indeed represent the standard phenotypic presentation for the disease and can as such be considered true cases. Further, these dogs can be considered representative of the diagnosed population of dogs, showing the full spectrum of the disease, rather than a narrower group that might be included in other case-control studies focusing on an extreme phenotype.

It is expected that prevalence estimates for cAD based upon veterinary diagnosis are underestimated due to mild cases not being taken to the vets and lack of recognition/recording of certain signs as being indicative of cAD^1^. Here, by collecting data directly from owners the cAD-RQ has shown its strength. Further evaluation of the questionnaire answers allowed us to identify two new sets of criteria within the cAD-RQ itself that were able to correctly classify 89–94% of diagnosed cases and exclude 99.9% of controls. The specificity of these criteria mean that 29–31% of dogs identified by them did not, at the time of the survey, have a diagnosis of cAD. However, investigations of the clinical signs reported by the owners of this group of ‘false positives’, referred to here as Potential Cases, revealed that they presented very similarly to diagnosed cases, if less severely, and very differently from Other non-cases and controls. It is highly plausible therefore to propose that these ‘false positives’ could in-fact be considered dogs with an allergic skin disease that have thus far gone undiagnosed and that they truly are potential cases. Reasons for this could be varied, some owners reported in the free text that their dog had only just started showing signs of skin problems and were currently undergoing veterinary exploration, others that their dogs skin problems didn’t impact the dog’s quality of life or had been considered too mild to warrant veterinary attention. The implications of this are vast when considering future management options for the disease, especially where breeding programmes are concerned, as many dogs may have the condition, but may lack an official diagnosis. Tools such as the questionnaire proposed here could help to identify such dogs. We envisage that the cAD-RQ and the associated diagnostic criteria presented here could be utilised in a clinical setting prior to clinical evaluation to capture skin health information relevant to diagnosis of cAD in a standardised manner. The criteria are suitable for use as a diagnostic aid only once clinical signs have begun to be noticed, so it would not be recommended that it be used to identify a dog as being ‘clear’ of allergies whilst it is still of an age where it is at risk (i.e. under 3 years of age).

A similar questionnaire to the cAD-RQ was presented in a recent study of Finnish dogs (published after this study had concluded) by Hakanen and colleagues^21^. They were able to show that allergy signs could be meaningfully combined to form an ‘allergy index’. In our study, a much higher proportion of dogs with a known diagnosis and Potential Cases exhibited allergy signs than did controls and ‘others’, yet allergy signs themselves weren’t included in the classification criteria. It is possible that an allergy index similar to that presented by Hakanen and colleagues could be used to further classify the severity of the cases and undiagnosed cases.

An important incidental ‘finding’ of this study was the strong willingness for dog owners to take part in this research, even when their dog had no skin problems. This allowed us to collect a large amount of skin health data in a relatively short period of time and, in conjunction with the strong construct validity of the questionnaire, acts as a proof-of-concept for the epidemiological evaluation of complex health conditions like cAD via owner report questionnaires.

Whilst this study only included purebred Labradors and Golden retrievers, it is anticipated that the tool could be used for any breed of dog due to the general nature of the criteria identified here and the focus on clinical signs common between breeds, which would allow for intra-breed comparisons. Previous diagnostic criteria have been shown not to be heavily impacted by breed variations^10^ although a validation study will need to be conducted to confirm this and make breed-specific adjustments to the cAD-RQ if necessary. The nature of this disease is too complex for any one tool or set of diagnostic criteria to be used in isolation. However, the questions presented here could be of value not only for researching cAD (in the broad sense) and associated skin conditions but for use in the veterinary setting as a method of collecting standardised data on the life history and clinical features of presenting dogs prior to a clinical evaluation. Recorded veterinary histories tend to be functional rather than detailed, and by using this questionnaire, detailed standardised data could be collected regarding skin health recorded directly by the owner prior to their consult; saving time in already time-pressured consultations and assisting in the diagnostic process.

## Methods

### Questionnaire development

A questionnaire for dog owners was developed to evaluate their dog’s skin health with respect to the clinical signs of cAD. Initial questions were asked of all respondents and were used to determine which further questions were asked. All participants were asked to record any skin-related diagnoses that had been made by a veterinarian, as well as any undiagnosed skin conditions. Participants were further asked whether their dog had now or in the past exhibited clinical signs related to pruritic skin conditions and were asked to score their dog on a modified Edinburgh pruritus scale^17^; modifications were minor wording changes and additional descriptions of itch related behaviour to further define the categories. Participants who answered that their dog had had no diagnosed skin problems or signs of skin disease were sent to the end of the questionnaire after completing the first page of the skin health questions. All other participants were asked further questions presented in an owner-friendly manner, relating to their dog’s skin condition, using question logic to ask questions only when relevant (i.e. based upon the owners answer to the previous question, see Supplementary Table S1 for all questions). These further questions were developed based upon dermatological literature regarding the clinical signs and diagnosis of cAD^11,15,22^. Additional questions were also asked regarding the demographics of the dog and living environment, which will be evaluated in a separate publication.

The questionnaire was piloted with owners of six atopic dogs from a local veterinary practice and feedback on the questions’ applicability, user-friendliness and clarity was requested to aid question refinement. Further refinement of the questions to aid user-friendliness was conducted via an in-depth review by researchers within the Centre for Evidence-based Veterinary Medicine (CEVM).

### Distribution

In order to limit the variability in the presentation of clinical signs and to gather a large enough number of responses to help validate the tool, for the purpose of this study only two of the UK’s most popular breeds were included; purebred Labrador and Golden retrievers. The final questionnaire (see Supplementary Table S1 and Supplementary Fig. S1 for all questions as they were trialled) was hosted on SurveyGizmo.com and could be accessed via a project specific website (www.itchydogproject.co.uk). The project was advertised through relevant media sources (i.e. Vet Record, the Vet Times and dog magazines), via social media (Facebook and Twitter), and was listed on The Dog Science Group webpage and The Kennel Club BARC site. Further recruitment was conducted via The Kennel Club, who sent direct emails out to registered owners of Labradors and Golden retrievers providing details of the project and inviting them to participate. All owners of these breeds were invited to participate no matter what the condition of their dog’s skin, with it being made clear that they did not have to have atopic dermatitis or itchy skin to take part. Owners of dogs aged less than 3 years of age with no skin conditions at all were excluded, due to the possibility that their dog may yet develop cAD so couldn’t be considered to be a control. If a dog had a skin condition it could participate at any age. Dogs of breeds other than purebred Labradors and Golden retrievers were excluded upon registration.

The online questionnaire was open for 4 months between March 11^th^ and July 10^th^ and the full dataset was downloaded on the 10^th^ of July as a CSV file for cleaning in Excel.

This project was conducted in accordance with the University of Nottingham’s Code of Research Conduct and Research Ethics and has been approved by the University of Nottingham, School of Veterinary Medicine and Science research ethics committee (identification reference 1979,170217). All potential participants were fully informed about the project and were provided with contact details to ask further questions or withdraw from participation. Written, informed consent to take part in the project and for the data to be used was gained upon registration for the project at the beginning of the questionnaire, without which they could not participate.

### Data cleaning

The data was cleaned by removing disqualified responses, where no consent was given, or the dog was a breed other than purebred Labrador or Golden retriever. Partial responses were compared against completed responses (using the columns containing the owners name and the dog’s name) to identify owners who had subsequently completed the questionnaire. Where this occurred, the partial response was removed.

Due to the nature of the ‘Date’ question in SurveyGizmo.com, people had the option to type in the date their dog was born, which led to great variation in the format the date took, and in many cases, left no way to distinguish whether they had used a British date format (dd/mm/yyyy) or US date format (mm/dd/yyyy). For this reason, exact ages at the time of survey completion could not be calculated, so the last two digits from every date of birth entry were selected to provide the year the dog was born. The year they were born was subtracted from ‘17’ to calculate the dog’s age to the nearest year.

The term ‘hot spots’ is often used by the public to refer to wet eczema, whilst ‘acute moist dermatitis’ is the veterinary terminology. Where owners did not select ‘wet eczema’ under skin conditions but went onto report their dog had hot spots or acute moist dermatitis in text boxes associated with the questions “Another skin or ear disease not listed here” or “Undiagnosed skin problems”, they were classified as having had wet eczema.

All checkbox answer options were dummy coded within Excel so that a selected answer was scored with a ‘1’ and an unselected answer was scored as a ‘0’. Any questions that had NA as an option were recorded as NA.

### Statistical analysis

Skin health data was analysed using SPSS v.22 (SPSS Inc., Chicago, IL). Dogs were first classified as a Case, Control or Other using a two-step process according to the logic outlined in Supplementary Table S1. For dogs in the Other group, further skin conditions were identified from information provided in the free text boxes when owners selected that their dogs had either “Another skin or ear disease not listed here” or “Undiagnosed skin problems”.

A new set of diagnostic criteria was identified from the questionnaire itself using simulated annealing. Simulated annealing is a data reduction technique that allows the selection of the most relevant subset of predictors from a much larger set of predictors in predictive modelling^23^. For large datasets, predictors can be redundant and noisy, and using the whole dataset can lead to overfitting and hence poor predictions. Therefore, reduction of the number of predictors also known as feature selection is an important step in predictive modelling. Several different heuristic optimisation algorithms exist that can find a subset of features that minimise the prediction errors. Here, the error function selected to minimise was based on the performance of the classification defined as: 1- overall accuracy. The definition of accuracy used was:

$$Accuracy=\frac{TP+TN}{TP+TN+FP+FN}$$

*[TP: true positive, TN: true negative, FP: false positive, FN: false negative]*

Accuracy and performance used to produce the cost functions was obtained using a random forest classifier. The partitioning of the classifier was performed using a 5-fold cross validation: the dataset was divided into 5 subsamples, where 4 subsamples were used for training and 1 subsample for validation. The process was then repeated 5 times until each of the 5 subsamples were used once for validation. The cost of the classifier was computed using the average cost function for all the 5 subsamples. A series of question subsets from 5 to 24 were selected, with the maximum number of iterations set to 30, the maximum number of sub iterations set to 10 and the initial temperature set to 10 with a temperature reduction rate of 0.99. The code for the simulation annealing procedure was written in Matlab R2017a (MathWorks Inc.).

Following the simulated annealing procedure, each of the predictors in the subsets obtained were tested for significance against a case/non-case using a Fisher test and the number of times each predictor appeared in a set of features was calculated. The most relevant features were considered to be those that appeared in 5 or more feature lists obtained through simulation annealing, and that were significant to p < 0.05 in the Fisher test. In this way a list of questions that could be used to predict diagnosis of cAD was selected. Since all questions were binary summed scores were tested for classification performance on the whole dataset of 4,111 dogs. Using the summed score on the entire population enabled us to isolate potential undiagnosed cases of atopic dermatitis (‘false positives’) from the group of dogs with ‘Other’ skin conditions creating four groups for comsparion^24^:

1. Cases: dogs whose owners reported a veterinary diagnosis of “atopic dermatitis or atopy (environmental allergies, including mite allergies)”
2. Controls were selected based upon the following criteria:
  - No reported diagnosis of “atopic dermatitis or atopy (environmental allergies, including mite allergies)”
  - No “current or past areas of abnormal skin (i.e. red, patchy, hairless, rough, swollen or discoloured)”
  - No “Current or past signs of abnormal itchiness (frequent and recurrent rubbing, licking, chewing or scratching of the same areas)”
  - Finally, dogs were excluded as a control if owners reported that the dog had an undiagnosed skin problem and the free text description mentioned allergies to plants, dust, household products, or seasonal itching.
3. Potential Cases: dogs without a current veterinary diagnosis of canine atopic dermatitis that get classified as cases by the summed score
4. Other: dogs with skin problems that were classified as a non-case by the summed score.

## Supplementary information

Supplementary materials

## Data Availability

Anonymised datasets analysed during the current study are available from the corresponding author on reasonable request.
