# Supplementary material for: Development and validation of a new standardised data collection tool to aid in the diagnosis of canine skin allergies
Source: Sci Rep. 2019 Feb 28;9:3039. doi: 10.1038/s41598-019-39630-3 (PMC6395597; doi:10.1038/s41598-019-39630-3)
Supplement: Supplementary file 1 — Supplementary materials [file 41598_2019_39630_MOESM1_ESM.docx]

SUPPLEMENTARY MATERIAL

Development and validation of a new standardised data collection tool to aid in the diagnosis of canine skin allergies

Harvey, N.D. ^1^, Shaw, S. ^1, 2^, Blott, S. C. ^1^, Vazquez Diosdado, J.A. ^1^ and England, G.C.W. ^1^

^1^ School of Veterinary Medicine and Science, The University of Nottingham, Leicestershire, United Kingdom

^2^ UK Vet Derm, 16 Talbot Street, Whitwick, Leicestershire, United Kingdom

Table S1. Questions asked in the cAD-RQ. * Indicates questions that were only shown depending on the answers provided to the previous question.

| **Registration Questions** | **Answer options** |
| --- | --- |
| Breed | Labrador |
|  | Golden retriever |
|  | Labrador cross |
|  | Golden retriever cross |
|  | Other |
| Coat colour (Labrador only)* | Yellow (all shades including Fox Red, White or Fawn) |
|  | Black |
|  | Chocolate |
|  | Silver |
| Sex of dog | Male/Female |
| Dogs D.O.B | (Calendar choice or Free text) |
| Kennel Club registered | Yes/No |
| Country dog lives | (Mass dropdown) |
| Where did you hear about the project? | Kennel Club/Breed Club |
|  | Facebook |
|  | Twitter |
|  | Vet |
|  | Guide Dogs |
|  | The Dog Science Group |
|  | A dog/pet magazine |
|  | Radio/TV |
|  | Word of mouth |
|  | Other |
| Type of dog | Pet |
|  | Guide dog or former guide dog |
|  | Other working/service dog |
|  | Gundog |
|  | Show dog |
|  | Breeding dog |
|  | Other |
| Consent to register | Yes/No |
| **Skin health questions** | **Answer options** |
| Has [dog's name] ever received a diagnosis from a veterinarian for any of the following skin problems (please tick all that apply)? | No skin related diagnosis |
|  | Atopic dermatitis or atopy (environmental allergies, including mite allergies) |
|  | Food allergies |
|  | Mange (i.e. scabies or demodex) |
|  | Yeast skin infection (often called Malassezia dermatitis) |
|  | Bacterial skin infection (often called pyoderma) |
|  | Flea allergic dermatitis (allergy to flea bites) |
|  | Ear infection/inflammation (Otitis externa) |
|  | Wet eczema |
|  | A skin or ear disease but I don't know which |
|  | Another skin or ear disease not listed here - Write In (Required) |
|  | I don't know this dog's veterinary history |
| Has [dog's name] ever had any undiagnosed skin problems in the past? | Yes/No |
| Please provide details of the undiagnosed skin problems and what you did to resolve them.* | (Free text) |
| Has [dog's name] ever been allergy tested (i.e. allergy blood test and/or skin allergy test) to help with diagnosis and treatment of allergies?* | Yes/No |
| If known, please provide details of what the test indicated [dog's name] is allergic to* | (Free text) |
| Does [dog's name] currently have (or have they in the past had) areas of abnormal skin (i.e. red, patchy, hairless, rough, swollen or discoloured)? | Yes/No |
| Does [dog's name] currently show (or have they in the past shown) signs of abnormal itchiness (frequent and recurrent rubbing, licking, chewing or scratching of the same areas)? | Yes/No |
| Did your vet provide or suggest a diet trial as part of the investigation of skin disease?* | Yes/No/Not applicable |
| Did or does [dog's name] frequently and recurrently display any of the following (please select all that apply)? | Scratching |
|  | Paw licking/chewing |
|  | Licking, biting or chewing other areas |
|  | Face rubbing |
|  | Sneezing |
|  | Runny nose |
|  | Watery eyes |
|  | None of the above |
| Please select the option that best describes [dog's name]'s itching when at its worst: | Normal grooming behaviour - perhaps a small scratch when waking or when groomed or treated with flea products, but otherwise little to see. |
|  | Occasional episodes of itching - notice scratching/rubbing/licking/chewing on several occasions throughout the day |
|  | More frequent itch - scratching/rubbing/licking/chewing is seen more frequently than expected, but itching stops when dog is sleeping, eating, playing or otherwise distracted. |
|  | Moderately increased itch - chewing and biting are seen regularly or scratching is seen throughout the day. Dog can be distracted from itch by play, food or voice. |
|  | Prolonged irritation when awake – dog regularly wakes up because of itching, or scratches in its sleep. It is difficult to distract dog from scratching/biting/chewing activity. Dog will scratch following mild skin manipulation. |
|  | Almost continuous itching - dog is scratching/chewing/biting throughout day and does not stop when distracted. Even in the vets, dog need to be physically restrained from scratching. |
| Does (or has) [dog's name] suffer(ed) from recurring digestive/gastrointestinal problems (such as diarrhoea, soft stools, jelly in stool)? | Yes/No |
| How often does your dog have such problems?* | Multiple times a week |
|  | Approximately once a week |
|  | Every few weeks |
|  | Every few months or less often |
| Does (or has) [dog's name] suffer(ed) from recurring vomiting? | Yes/No |
| How often does your dog vomit?* | Multiple times a week |
|  | Approximately once a week |
|  | Every few weeks |
|  | Every few months or less often |
| Does your dog receive flea and/or tick control? | No flea/tick control |
|  | Frontline spot-on |
|  | Frontline Combi Spot-on |
|  | Advantage Spot-on |
|  | Advantix Spot-on |
|  | Advocate Spot-on |
|  | Clear (Bob Martin) Spot-on |
|  | Endectrid Spot-on |
|  | Stronghold Spot-on |
|  | Comfortis tablets |
|  | Nexgard or Nexgard Spectra tablets |
|  | Bravecto tablets |
|  | Simparica tablets |
|  | Johnson 4Fleas tablets |
|  | Program tablets |
|  | Seresto collar |
|  | Other - Write In (Required) |
| If you have any comments about flea/tick control (i.e. why you don't use it, what alternatives you use, or why you use a specific type) please use this space to record them | (Free text) |
| Has [dog's name] had an injury or health problem in the past three months (other than any skin problems already covered)? | Yes/No |
| Please describe the injury or health problem | (Free text) |
| **Skin health: continued *(those classified as Controls ended the survey here & were sent to the end, all others answered the following questions)*** | |
| Please indicate which areas of your dog's body they scratch, lick, chew or rub frequently when symptomatic by selecting from the images below (select all that apply). The areas in red indicate the affected area:  (see Fig. S1 for examples of the images used here) | Front paws |
|  | Back paws |
|  | Muzzle |
|  | Ears |
|  | Chin |
|  | Underbelly - tummy and inner thighs |
|  | Armpit area |
|  | Bottom/base of tail |
|  | Elbows |
|  | Around eyes |
|  | Collar region |
|  | Back |
|  | None of these areas |
| Please use this space to add any comments you have about the location of your dog's skin problems, for example if the problem occurs around the area of a wound. | (Free text) |
| Please select all options that apply to [dog's name]'s skin when it is as its worst. In certain areas my dog's skin is: | Reddened |
|  | Damaged or broken by scratching/licking/chewing |
|  | Bald or has thinned fur |
|  | Rough and scaly to the touch |
|  | Darkened in colour |
|  | Swollen |
|  | Moist and weeping |
|  | Thickened |
|  | Greasy to the touch |
|  | None of these |
| Thinking about the previous question, please indicate all of the places where your dog's skin is affected (click all that apply):*  (see Fig. S1 for examples of the images used here) | Front paws |
|  | Back paws |
|  | Muzzle |
|  | Inside of ears (not edges) |
|  | Edge of the ear flap |
|  | Chin |
|  | Underbelly - tummy and inner thighs |
|  | Armpit area |
|  | Bottom/base of tail |
|  | Elbows |
|  | Around eyes |
|  | Collar region |
|  | Back |
|  | None of these areas |
| How old was your dog when you first noticed their skin problems? | Between 0-6 months |
|  | Between 7-12 months |
|  | 1 year old |
|  | 2 years old |
|  | 3 years old |
|  | 4 years old |
|  | 5 years or older |
|  | I don't know |
|  | Not Applicable |
| Does [dog's name]’s skin improve or worsen in certain seasons? Please choose the most appropriate answer: | It stays the same all year round |
|  | It is worst in the Summer |
|  | It is worst in the Winter |
|  | It is worst in Spring and/or Autumn |
|  | Has good and bad spells, but no obvious seasonal pattern |
|  | Not applicable |
| Does [dog's name]’s skin problem improve (even temporarily) when treated with steroidal medication (anti-inflammatory treatment)? These could include: creams (e.g. Fuciderm or Isaderm), steroid sprays (e.g. Cortavance), steroid tablets (e.g. Prednisolone/Prednicare or Methylprednisolone/Medrone) or steroid injections. | Yes |
|  | No |
|  | Not tried |
|  | Not applicable |
| Does [dog's name]’s skin problem improve in response to other prescribed medications, such as oclacitinib (Apoquel) tablets, ciclosporin (e.g. Atopica or Cyclavanceand), immunotherapy (e.g. hyposensitisation or allergy vaccines) or anti-histamines such as Piriton or chlorphenamine? | Yes |
|  | No |
|  | Not tried |
|  | Not applicable |
| Does [dog's name]'s skin improve on an exclusion diet? | Yes |
|  | No |
|  | Not tried |
|  | Not applicable |
| What types of treatment (veterinary medication, supplements or management regimens) have you found most beneficial for managing [dog's name]'s skin problems? | (Free text) |
| Do any of [dog's name]’s relatives (parents or siblings) also have the same or similar skin conditions? | Yes/No/I don't know |


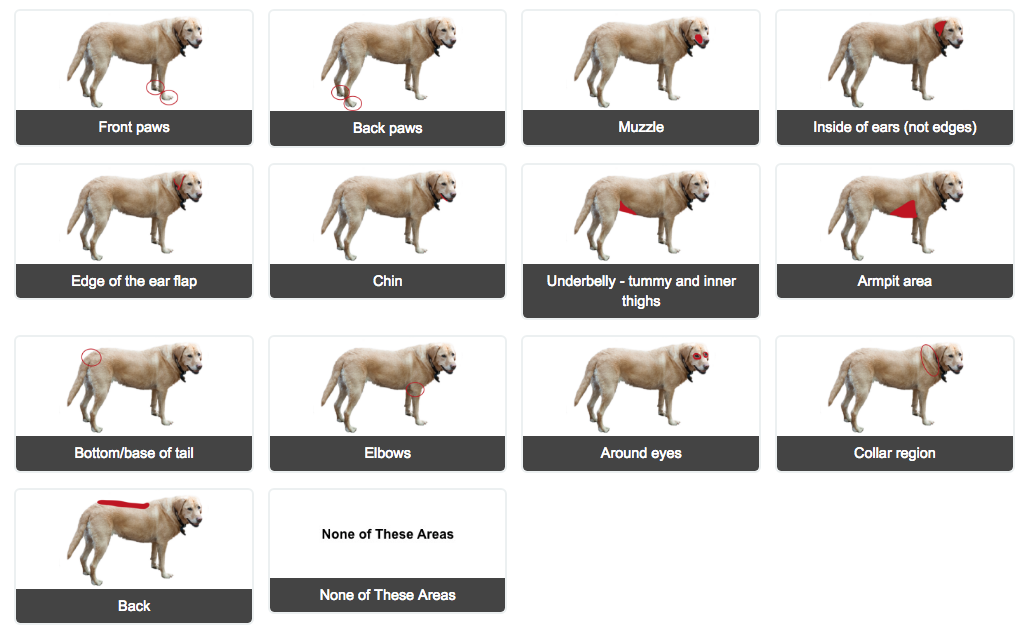


**Figure S1.** Images that accompanied body area descriptions.

Table S2 Demographic details of all individual, eligible dogs based on information provided during registration for the project (n=4,111). *Note*: for ‘Type of dog’ one dog could be in multiple categories, for example being both a ‘Pet’ and a ‘Gundog’ or a ‘Show dog’ and a ‘Breeding dog’.

| **Registration Questions** | **Answer options** | **N** |
| --- | --- | --- |
| Breed | Labrador | 2,803 |
|  | Golden retriever | 1,308 |
| Coat colour (Labrador only) | Yellow (all shades including Fox Red, White or Fawn) | 770 |
|  | Black | 1,239 |
|  | Chocolate | 497 |
|  | Silver | 1 |
| Sex of dog | Male | 2,100 |
|  | Female | 2,011 |
| Kennel Club registered | Yes | 3,628 |
|  | No | 353 |
| Where did you hear about the project? | Kennel Club/Breed Club | 2,544 |
|  | Facebook | 624 |
|  | Twitter | 18 |
|  | Vet | 37 |
|  | Guide Dogs | 8 |
|  | The Dog Science Group | 18 |
|  | A dog/pet magazine | 8 |
|  | Radio/TV | 1 |
|  | Word of mouth | 81 |
|  | Other | 64 |
|  | Email (origin not given) | 267 |
|  | (blank) | 373 |
| Type of dog | Pet | 3,452 |
|  | Guide dog or former guide dog | 20 |
|  | Other working/service dog | 42 |
|  | Gundog | 339 |
|  | Show dog | 129 |
|  | Breeding dog | 121 |
|  | Other | 102 |
| Country dog lives in | United Kingdom | 3770 |
|  | United States | 186 |
|  | Canada | 30 |
|  | Ireland | 21 |
|  | France | 15 |
|  | Australia | 12 |
|  | Germany | 12 |
|  | Netherlands | 9 |
|  | Spain | 6 |
|  | Portugal | 5 |
|  | Switzerland | 5 |
|  | Bermuda | 4 |
|  | Sweden | 4 |
|  | Afghanistan | 3 |
|  | Mexico | 3 |
|  | Belgium | 2 |
|  | Czech Republic | 2 |
|  | Italy | 2 |
|  | United Arab Emirates | 2 |
|  | Brazil | 1 |
|  | China | 1 |
|  | Croatia | 1 |
|  | Denmark | 1 |
|  | Hong Kong | 1 |
|  | Hungary | 1 |
|  | India | 1 |
|  | Jamaica | 1 |
|  | Malaysia | 1 |
|  | New Zealand | 1 |
|  | Norway | 1 |
|  | Pakistan | 1 |
|  | Romania | 1 |
|  | Russia | 1 |
|  | Singapore | 1 |
|  | South Africa | 1 |
|  | Turkey | 1 |


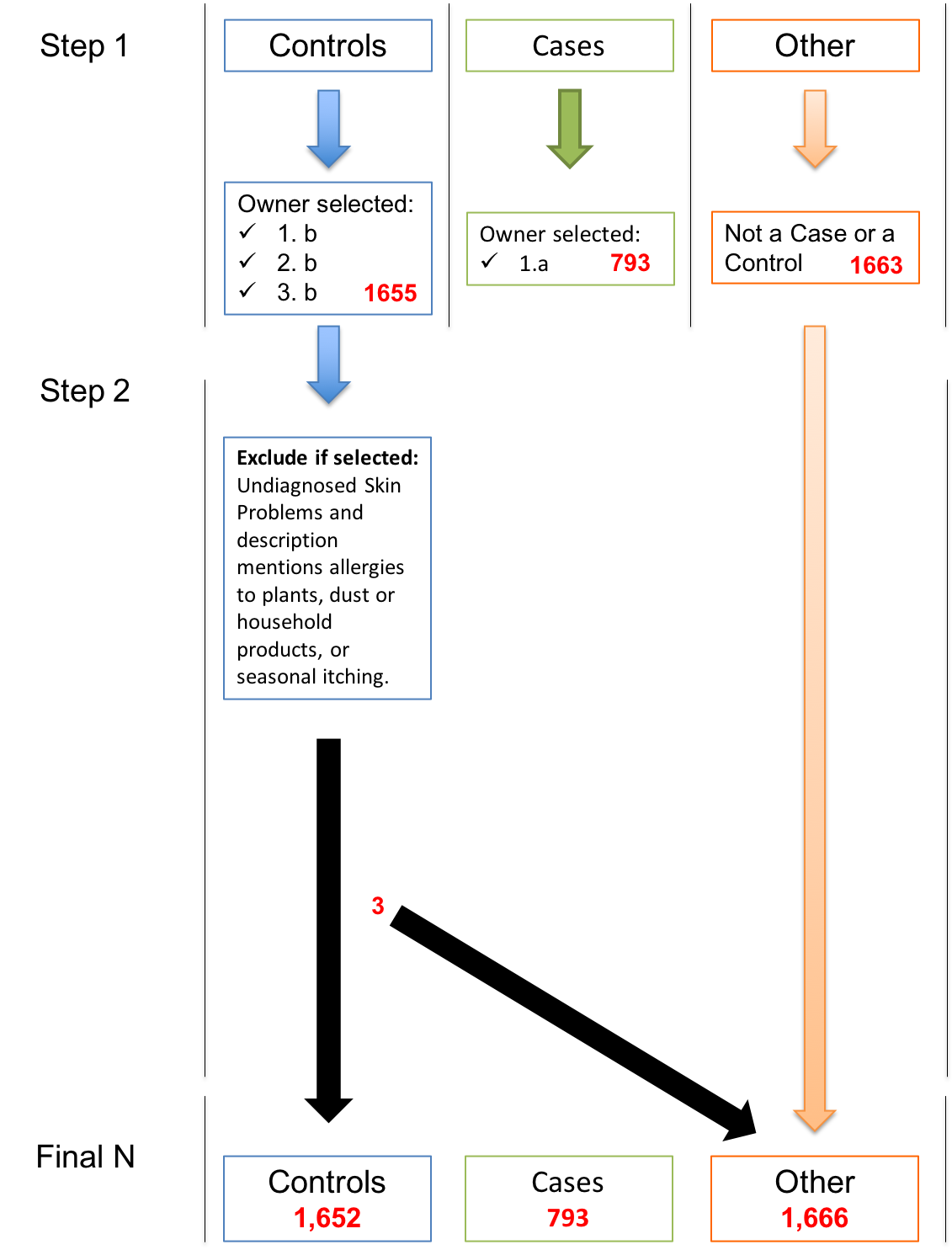


Figure S2. Flowchart depicting the process used to designate dogs as Cases, Controls, and Other.

**Table S3.** Questions from the cAD-RDQ that were used in determining Cases and Controls as indicated in Figure S1. *Cases* = owner selected 1.a. *Controls* = owner selected 1.b + 2.b + 3.b, but were excluded and classified as a potential case if they had selected the dog had an undiagnosed skin problem and the free text description mentioned allergies to plants, dust, household products, or seasonal itching.

| Part 2 – Your dog's skin | Answer options |  | Logic ID |
| --- | --- | --- | --- |
| Veterinary diagnoses of | Atopic dermatitis or atopy (environmental allergies, including mite allergies) |  | 1.a |
|  | No skin related diagnosis |  | 1.b |
| Current or past areas of abnormal skin (i.e. red, patchy, hairless, rough, swollen or discoloured) | Yes |  | 2.a |
|  | No |  | 2.b |
| Current or past signs of abnormal itchiness (frequent and recurrent rubbing, licking, chewing or scratching of the same areas) | Yes |  | 3.a |
|  | No |  | 3.b |


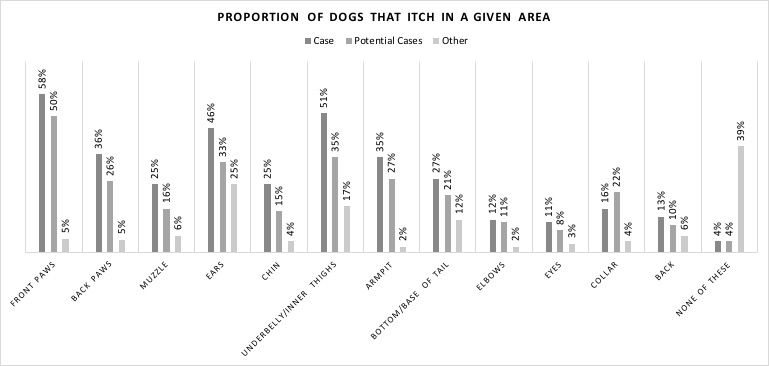


Figure S3 Proportion of dogs in each problem skin group that exhibited itchy skin in each body area.

**Table S4.** Logistic regression results comparing Cases and Others to False Positives for frequency of different skin abnormalities (lesions).

|  | **Case** | | | **Other** | | |
| --- | --- | --- | --- | --- | --- | --- |
|  | **p** | **OR** | **95% CI** | **p** | **OR** | **95% CI** |
| Reddened | **<0.001** | **2.09** | **1.72-2.54** | **<0.001** | **0.31** | **0.25-0.39** |
| Damaged/broken | **<0.001** | **2.42** | **1.99-2.96** | **<0.001** | **0.22** | **0.16-0.30** |
| Bald/thinned fur | **<0.001** | **1.76** | **1.45-2.13** | **<0.001** | **0.18** | **0.14-0.24** |
| Rough/scaly | **0.001** | **1.45** | **1.17-1.79** | **<0.001** | **0.31** | **0.22-0.42** |
| Darkened | **<0.001** | **1.94** | **1.53-2.46** | **<0.001** | **0.3** | **0.20-0.44** |
| Swollen | **<0.001** | **1.79** | **1.34-2.39** | **<0.001** | **0.34** | **0.21-0.55** |
| Moist/weeping | **<0.001** | **1.59** | **1.25-2.02** | **<0.001** | **0.53** | **0.39-0.74** |
| Thickened | **0.027** | **1.4** | **1.04-1.89** | **<0.001** | **0.23** | **0.13-0.41** |
| Greasy | 0.119 | 1.39 | 0.92-2.11 | **0.023** | **0.50** | **0.28-0.91** |
| None of these | **<0.001** | **0.42** | **0.32-0.55** | **<0.001** | **5.24** | **4.19-6.56** |


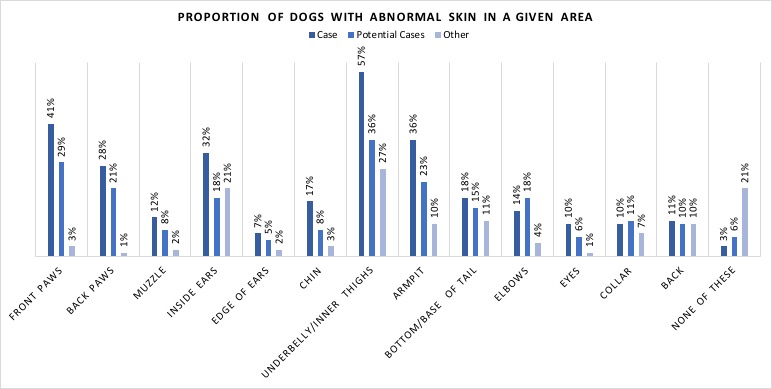


Figure S4 Proportion of dogs in each problem skin group that exhibited abnormal skin broken down by affected body area.

**Table S5.** Logistic regression results comparing Cases and Others to False Positives for location of skin lesions.

|  | **Case** | | | **Other** | | |
| --- | --- | --- | --- | --- | --- | --- |
|  | **p** | **OR** | **95% CI** | **p** | **OR** | **95% CI** |
| Front paws | **<0.001** | **1.66** | **1.33-2.06** | **<0.001** | **0.06** | **0.03-0.14** |
| Back paws | **0.001** | **1.50** | **1.18-1.91** | **<0.001** | **0.04** | **0.01-0.14** |
| Muzzle | **0.013** | **1.55** | **1.09-2.20** | **0.001** | **0.18** | **0.06-0.49** |
| Inside of ears | **<0.001** | **2.16** | **1.69-2.76** | 0.272 | 1.22 | 0.86-1.73 |
| Edge of ears | 0.061 | 1.51 | 0.98-2.32 | 0.064 | 0.44 | 0.18-1.04 |
| Chin | **<0.001** | **2.51** | **1.79-3.49** | **0.019** | **0.43** | **0.21-0.87** |
| Underbelly/inner thighs | **<0.001** | **2.32** | **1.88-2.87** | **0.008** | **0.66** | **0.48-0.89** |
| Armpit | **<0.001** | **1.92** | **1.53-2.41** | **<0.001** | **0.35** | **0.23-0.55** |
| Bottom/base of tail | 0.142 | 1.23 | 0.93-1.62 | 0.107 | 0.70 | 0.45-1.08 |
| Elbows | **0.038** | **0.74** | **0.56-0.98** | **<0.001** | **0.18** | **0.09-0.34** |
| Eyes | **0.015** | **1.62** | **1.09-2.38** | **0.003** | **0.17** | **0.05-0.56** |
| Collar region | 0.324 | 0.84 | 0.60-1.18 | **0.046** | **0.58** | **0.34-0.99** |
| Back | 0.413 | 1.15 | 0.82-1.61 | 0.798 | 1.06 | 0.67-1.69 |
| None of these | **0.006** | **0.46** | **0.27-0.80** | **<0.001** | **4.45** | **2.91-6.82** |
